# Implications of Vascularized Composite Allotransplantation in Plastic Surgery on Legal Medicine

**DOI:** 10.3390/jcm12062308

**Published:** 2023-03-16

**Authors:** Valentin Haug, Adriana C. Panayi, Samuel Knoedler, Sina Foroutanjazi, Martin Kauke-Navarro, Sebastian Fischer, Gabriel Hundeshagen, Yannick Diehm, Ulrich Kneser, Bohdan Pomahac

**Affiliations:** 1 Department of Hand-, Plastic and Reconstructive Surgery, Microsurgery, Burn Trauma Center, BG Trauma Center Ludwigshafen, University of Heidelberg, 69120 Ludwigshafen, Germany; 2 Division of Plastic Surgery, Department of Surgery, Brigham and Women’s Hospital, Harvard Medical School, Boston, MA 02115, USA; 3 Division of Plastic and Reconstructive Surgery, Yale University School of Medicine, New Haven, CT 06520, USA

**Keywords:** vascularized composite allotransplantation, facial recognition, biometrics, DNA, chimerism, fingerprint identification

## Abstract

Background: When a patient receives a transplant—be it classically an organ or bone marrow or, more recently, composite allotransplantations of the limb or face—it can result in artificial chimerism. Such chimerism raises considerations in forensic medicine, a field that relies on the collection and identification of biological samples from crime scenes. Beyond this chimerism, composite allotransplantations create further challenges. Methods: After screening the literature and press releases, we provide a brief history and summary of some of the technologies used in forensic identification, explaining their advantages and pitfalls in the light of transplantation and cautioning against misidentifying those who evade justice by taking advantage of such considerations. Results: With face transplantation, patients can receive the skin, hair, salivary glands, teeth, and oral and nasal mucosa of their donors, components which hold great importance in forensic science. Modern technologies such as computer-assisted facial recognition, although gradually becoming more accurate over time, also face new challenges in this post-transplantation era as facial recognition software can be misled by surgical alterations of the face or face transplantation. With limb transplantation, there is an impact on fingerprint identification. Conclusions: Both surgical transplantation techniques and forensic technologies have seen incomprehensibly great innovation in the past century. Given the growing rate of successful composite transplantation in the USA and worldwide, it is now important for law enforcement agents to be aware of the new possibility of having two sets of genetic material, hair, saliva, fingerprints, or even facial recognition data for the same individual.

## 1. Introduction

A classic Hollywood storyline, particularly during the Film Noir era of the 1940s and 1950s, is law evasion through facial modification. Plastic surgeons were often depicted as altering the outer appearance of criminals so extensively—sometimes even through face transplantation—that it would allow gangsters to acquire new identities [1]. For decades, these tales were simply implausible scenarios conjured up from vivid imaginations. In 2018, however, during the notorious “El Chapo” trial, this scenario became a reality. The Colombian drug lord Juan Carlos Ramirez Abadia testified in court that he had, for years, successfully evaded arrest by undergoing extensive facial modification. His facial structure was so heavily altered that it rendered facial recognition software inadequate—ultimately, voice recognition technology had to be employed for his arrest. Ever since the first facial transplant in 2005, the field has been growing rapidly, and current surgical skills and technologies make complete facial modification possible. Therefore, the forensic and legal ramifications of these achievements can no longer be ignored.

In a 2019 study, Sanz-Piña et al. reviewed the literature and concluded that hematopoietic stem cell transplantation can result in chimerism, the possession of cells with more than one set of genetic markers. This can prove challenging for legal–medical experts [2]. The authors explored the impact that transplantation may have on the genetic analysis of biological samples collected from crime scenes, including hair, blood, and skin, among others. With advancements in forensic science and technology, investigators are no longer limited to biological samples [3]. Newer technologies, such as facial recognition, are just as widely employed as older technologies such as fingerprint analysis and dental record analysis.

The face and hand transplant recipient community are of special interest within this context, as a major source of forensic biological samples is dependent on the orofacial structure and upper extremities. Therefore, in this paper, we set out to discuss the different types of forensic technologies that are currently in use, including biological sample analysis, facial and anthropomorphic feature analysis, and fingerprint analysis, exploring these techniques specifically within the context of vascularized composite allotransplantation (VCA) in order to highlight limitations as well as special considerations. This study is exempt from ethical approval.

## 2. Biological Sample Analysis and Artificial Chimerism in VCA

Genetic analysis has become efficient with technological advancement and is currently one of the most widely employed forensic techniques. Small variations in DNA segments, such as single nucleotide polymorphisms (SNPs) and short tandem repeats (STRs), allow for unique intraspecies traits and genetic and phenotypic diversity. The analysis of SNPs and STRs has been employed in forensics for over 20 years and has been particularly useful in comparative studies in which suspects are already known to law enforcement. More recently, familial searching, which is based on similar alleles shared between biological relatives, has been used for identification. While DNA analysis provides a more in-depth picture, mitochondrial DNA (mtDNA) analysis, which helps maternal ancestry tracing, can be utilized when nuclear DNA is unavailable [4].

Genetic information can be extracted from a variety of sources and used as a possible source of identification, such as DNA from fingernail clippings present at the site of a crime [5]. Owing to the durability of their structure, teeth and bones provide protection for DNA and are commonly used for identification in case of degraded or fragmented human remains [6]. Due to its methylation pattern, DNA is more stable than mRNA. Detecting this methylation pattern using DNA microarrays has been proposed as a method for detecting the body fluid type [7]. Dried saliva samples can be analyzed via Raman spectroscopy, and their chemical composition can be distinguished from dried semen or blood [8]. Using the same technique, the biological sex or race of the person of interest can be predicted from blood and saliva [9]. It should be noted that blood- and saliva-specific mRNA markers can remain stable for many years and may be used for the detection of the origin of tissue [10]. It is predicted that DNA samples from crime scenes may be used in the near future to predict externally visible characteristics of a person of interest; this may be useful in identifying those whose information is not available in current databases [11].

There are disadvantages to these methods, however. Environmental factors can facilitate specimen degradation and may affect how reliably the DNA can be extracted from a crime scene [6]. Further, PCR amplification of the genetic contaminants at the scene can lead to false results [5]. Similarly, mtDNA heteroplasmy, paternal leakage, and recombination are possible factors affecting the accuracy of mtDNA extraction in forensic identification [12]. According to a study, failure of forensic sciences, especially DNA studies, result in a large majority of wrongful convictions, second only to erroneous eyewitness identification. This error is made in the binary interpretation of DNA studies rather than seeing them from a probabilistic view and failing to accept the possibility of error in such studies [13]. Chimerism is another confounding factor in genetic profiling, leaving the identification of the biological vestiges ambiguous.

**Figure 1:**
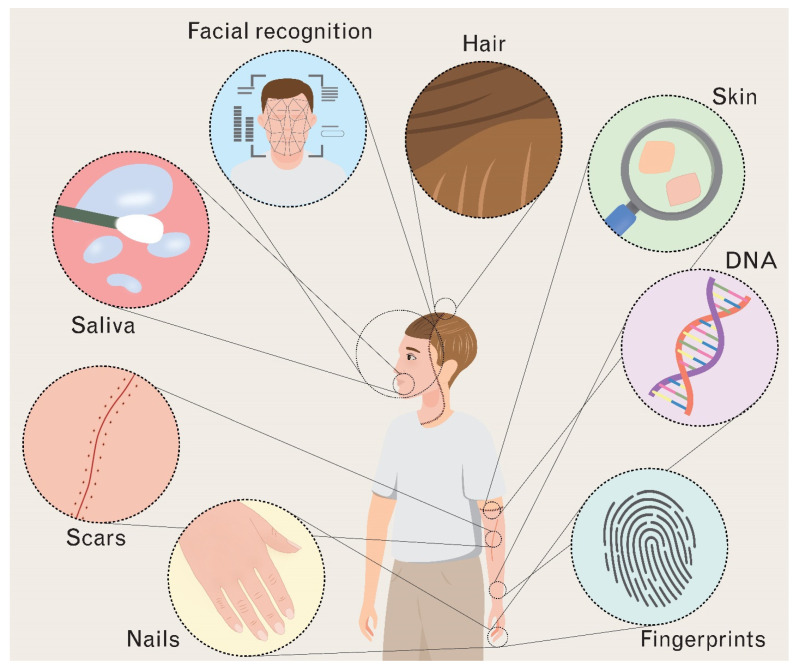
The face and hand transplantation recipient as a potential challenge for forensic methodology.

Through the lens of forensic genetic analysis, VCA is associated with two main challenges:

(i) Naturally, any allogeneic transplantation involves the transfer of foreign, non-autologous tissue. In VCA, the transplanted units comprise a heterogenous set of tissues including skin, bone, muscle, hair, and vasculature (Figure 1). Accordingly, as part of VCA surgery, patients receive the donor’s biological and miscellaneous material. Therefore, postoperatively, VCA patients carry tissue from two separate identities. In addition, transplant patients may also receive facial or scalp hair from donors during VCA surgery. Microscopic hair analysis is not very accurate to begin with and can lead to false results given the intrapersonal variations between scalp, pubic, scrotal, and labial hair [4]. Briefly, depending on the origin of the analyzed sample and tissue from VCA recipients, forensic identification may yield different (and false) results;
(ii) Previous studies demonstrated that allogeneic hematopoietic stem cell transplantation can result in the coexistence of donor cells and host cells with different genomes (i.e., chimerism) [14,15]. Such stem cells are typically isolated from bone marrow [16]. The component of a vascularized bone marrow compartment (often the radius, ulna, or jaw are included) is unique to VCA. Accordingly, there is a mounting body of evidence pointing toward the development of chimerism as a passenger within VCA surgery. In animal VCA models, macrochimerism has been well documented [17,18,19,20]. Yet, clinically, the correlation between VCA and chimerism remains to be fully elucidated. While Granger et al. and Kanitakis et al. were able to observe microchimerism in human hand allografts, Schultz et al. detected both donor and host DNA in samples of human full-facial transplants [21,22]. Notably, in all three cases, the microchimerism was found to be of transient nature, with all donor cells vanishing within the first postoperative year. Given the extremely low levels of donor-derived cells/DNA in the host organism, (micro)chimerism analysis in human VCA necessitates highly discriminative and efficient techniques. Further long-term studies are needed to thoroughly investigate the development and frequency of (stable) chimerism in clinical VCA.

VCA is gradually paving its way into clinical routine. Due to the rising number of VCA patients, it is essential to consider the biochemical characteristics of VCA samples from a forensic perspective. In fact—given the risk of chimerism—the vestiges of VCA patients may not serve as reliable sources for personal identification or paternity testing. Particular attention should be paid to saliva, which is considered to be the largest source of DNA at crime scenes: in facial VCA, minor and major salivary glands and mucosal epithelium are often procured and transplanted [3]. Therefore, it is likely that saliva samples from the recipients may include their original genetic material, as well as the material obtained from their donors. Such chimerism may lead to false positive or negative results of the saliva or buccal swab specimen. Further, it remains unclear whether the donor cells also migrate outside the transplant in the host circulation and can, therefore, be detected in non-VCA tissue. Similarly, following hematopoietic stem cell transplantation, donor cells appeared in non-hematological samples such as sperm, hair follicles, and urine [23,24]. To date, the legal protections that exist are ill-suited to protect defendants from being prosecuted for unreliable identifications. In the field of forensic genetics, the theoretical possibility of VCA patients carrying more than one set of DNA must be accounted for in future analyses.

## 3. Facial Recognition and Anthropomorphic Studies

Facial recognition software involves advanced technologies, massive database comparisons, and complex computer algorithms that have increasingly become utilized in criminal investigations. The purely manual method of facial recognition heralds back to the late 1800s, when the French policeman, Alphonse Bertillon, utilized anthropometric measures, such as facial traits, mug shots, and body markings, for the first time to identify criminals. His work has been improved upon, and its variations are still used in forensics worldwide [25]. With the advent of artificial intelligence, technologies such as computer topographic imaging, coupled with RE/FACE computer software, have been developed to assist in the reconstruction of facial features based on skull morphologies and bony prominences [26].

Facial reconstruction has been shown to have good accuracy and efficiency, especially with regard to 2D models [27]. The camera-specific processing of images leaves intrinsic fingerprints that can help determine authenticity and indicate if an image has been further manipulated by other software, which is important in forensic identification [28]. While opponents of using facial recognition argue that such software violates individual privacy, a court in the UK recently ruled otherwise; furthermore, five major police departments in the USA have shown interest in using this technology, which is currently already in use by major technology companies such as Google and Facebook [29]. Soft biometrics such as ethnicity, gender, and facial marks which cannot, when used on their own, accurately identify a subject, can be used in combination with other technologies to improve recognition accuracy [30]. Most recently, the Federal Bureau of Investigation (FBI) has been working on adding facial recognition photographs, including scars, tattoos, and other skin marks, to the Next Generation Identification (NGI) database, which also contains data on iris scans, palm prints, and fingerprints; the NGI is accessible to all levels of national and international law enforcement [31].

Despite its notable advantages, 2D facial recognition is error-prone and even slight differences in orientation or facial expression can significantly alter results [32]. Furthermore, experts have argued that computer-assisted facial identification is not more efficient than manual methods as they support that accurate facial reconstruction using data from the skull and bony prominences is not yet possible as it is prone to subjective errors [27]. Resemblance rating, which is the comparison of the forensic facial approximation with the skull of the person of interest, has shown a lack of statistical significance in the detection of a target person [33]. Challenges associated with automated facial recognition are textural and structural changes due to aging, placing too much weight on facial marks, or a comparison with flawed or misleading forensic sketches [34]. Manual facial comparisons can be challenging given that the differences between a known subject and the suspect may be invisible to the human eye due to the quality of the image or distortions post-processing [30]. Another major issue with facial recognition is its dependency on consistent lighting conditions, leading to the risk of false identification as well as unequally affecting different ethnicities [31,35]. While the UK has ruled in favor of facial recognition technology, the EU has placed a temporary ban of five years on the use of facial recognition in order to “figure out a sound methodology for assessing the impacts of this technology and possible risk management measures”.

Improvements in surgical techniques have led to optimal, high-quality face transplantation outcomes which can render facial identification software inadequate. This is a challenge that is unique to this patient population, as their facial structure and appearance are significantly different before and after the transplantation [36]. It should be noted that commercially available facial recognition software is already in use in the field of face transplantation as it provides the ability to study motor and social function recovery after surgery [37]. A unique consideration in this relatively small population of people is that a majority of their post-transplant images are readily available on the internet for utilization by forensic investigators. Thus, until face transplant becomes normalized, recipients will have a publicly recognizable face that is easily found in an image search.

## 4. Fingerprint Analysis

Fingerprint analysis, which allows for identification based on a certain number of matching ridge characteristics, with 12 or more points providing the strongest evidence, has been used as an acceptable method of identification for over a century. The uniqueness of each fingerprint is widely accepted in part due to anatomical studies and anecdotal evidence [38].

Fingerprint recognition is generally considered to be a more accurate modality than facial recognition [39]. In addition to the ease of use, vast experience, and utilization of the technique, fingerprint sweat analysis also has the potential of identifying sex, which is particularly helpful in narrowing down a list of suspects [40].

On the other hand, fingerprint identification can be subject to bias and intentional manipulation, leading to false outcomes. Incidental similarities are inevitable and can cause erroneous identification [41]. Added pressure can induce fingerprint distortions, which can challenge identification accuracy based on a fingerprint map [42]. Contrary to previous beliefs, fingerprints are fluid and can change over time [38]. Since fingerprints are vulnerable to external damage, they can be altered accidentally—or intentionally to evade the justice system—through burning, cutting, chemical injury, surgery, and micro-implantation.

In addition to the intentional alteration of one’s fingerprints, a pitfall of using fingerprint analysis in the post-transplantation era refers specifically to hand transplant recipients who can have their donor’s fingerprints. Hence, as the number of hand transplantation recipients continues to increase, it is essential for law enforcement agencies to be aware of the possibility that suspects can have two sets of fingerprints. This caveat was also highlighted in a case report from Poland, which analyzed the fingerprints of a single hand transplant recipient over a 40-month post-transplant period. The donor’s fingerprints had been recorded in a criminal database and thus provided baseline/reference material. Throughout nine measurements at six-month intervals, no significant differences were found in the appearance of the minutiae and white lines, or in the distance between papillary ridges. Notably, Szajerka et al. reported that the number of solitary white lines was the highest at three months after transplantation—parallel to a suspected rejection episode. This finding may indicate that immunogenic rejection can modify the pattern of skin ridges. Therefore, long-term studies are needed to investigate to what extent chronic rejection in hand transplantation can interfere with the fingerprint and its microstructure [43]. Menna and Scarpis also described a case of a hand transplant recipient with two different fingerprints while underscoring the medicolegal implications of this identarian ambiguity: hypothetically, convicted and documented criminals who had undergone hand transplantation may commit a felony with the newly transplanted hand [44]. Subsequent fingerprinting would not yield any matches in the criminal police records, thus concealing the culprit’s real identity—with potentially drastic consequences. The vulnerability of fingerprint analysis in our high-tech era was further exemplified by the case of a Chinese woman who illegally entered Japan by having her fingerprints surgically transplanted [45,46]. According to the Japanese police, this biometric fraud may be a widespread practice that remains mostly undetected.

## 5. Forensic Odontology

The evaluation of dental evidence is a cornerstone in the medicolegal sphere. Data obtained from the oral cavity may help identify the human remains of victims. Forensic odontology is of particular interest when conventional identification methods, such as fingerprints and visual identification, cannot be applied to decomposed or skeletonized bodies [47]. In addition, buccodental studies can provide information about criminal proceedings through the evaluation of bite marks or child abuse [48]. Therefore, this interdisciplinary specialty of forensic sciences and stomatology plays a crucial role in the ante- and postmortem identification of victims and culprits [49]. The fundamental principles in the field of dental identification are based on the comparison of teeth and dental impressions. These, however, can be altered by orthognatic surgery in the case that patients suffer from dentomaxillofacial deformities or simply wish for an aesthetic improvement [50].

Depending on the patient’s defects, facial VCA can also include the transplantation of the mandible or maxilla with teeth [51]. In other words, during facial VCA surgery, the patient may receive a new denture with complete donor dentition and/or newly implanted osseointegrated teeth [52]. In such cases, the intraoral identity of the VCA patient changes, and any forensic analysis would falsely suggest the donor. Forensic and medical law experts must be aware of this in order to avoid misidentification.

Given the rarity of VCA, a central database including genetic information, fingerprints, etc., might suffice to address potential issues. We, however, acknowledge potential issues with doctor–patient confidentiality.

## 6. Summary and Conclusions

Both surgical transplantation techniques and forensic technologies have seen incomprehensibly great innovation in the past century, with science and forensics improving the rate of successful criminal investigations and face and hand transplantation raising new challenges. Suspects taking advantage of surgical techniques to evade the criminal justice system have become a reality. Given the growing rate of successful composite transplantation in the USA and worldwide, it is now important for law enforcement agents to be aware of this new possibility of having two sets of genetic material, hair, saliva, fingerprints, or even facial recognition data for the same individual.

## Data Availability

Not applicable.
